# Digital Literacy in the Medical Curriculum: A Course With Social Media Tools and Gamification

**DOI:** 10.2196/mededu.4411

**Published:** 2015-10-01

**Authors:** Bertalan Mesko, Zsuzsanna Győrffy, János Kollár

**Affiliations:** ^1^ Institute of Behavioural Sciences Semmelweis University Budapest Hungary

**Keywords:** medical education, social media, digital literacy

## Abstract

**Background:**

The profession of practicing medicine is based on communication, and as social media and other digital technologies play a major role in today’s communication, digital literacy must be included in the medical curriculum. The value of social media has been demonstrated several times in medicine and health care, therefore it is time to prepare medical students for the conditions they will have to face when they graduate.

**Objective:**

The aim of our study was to design a new e-learning-based curriculum and test it with medical students.

**Method:**

An elective course was designed to teach students how to use the Internet, with a special emphasis on social media. An e-learning platform was also made available and students could access material about using digital technologies on the online platforms they utilized the most. All students filled in online surveys before and after the course in order to provide feedback about the curriculum.

**Results:**

Over a 3-year period, 932 students completed the course. The course did not increase the number of hours spent online but aimed at making that time more efficient and useful. Based on the responses of students, they found the information provided by the curriculum useful for their studies and future practices.

**Conclusions:**

A well-designed course, improved by constant evaluation-based feedback, can be suitable for preparing students for the massive use of the Internet, social media platforms, and digital technologies. New approaches must be applied in modern medical education in order to teach students new skills. Such curriculums that put emphasis on reaching students on the online channels they use in their studies and everyday lives introduce them to the world of empowered patients and prepare them to deal with the digital world.

## Introduction

In the last couple of years, the Internet has played an increasingly important role in communication and information management in medicine and health care. An information-based society leads to an exponential increase in the amount of information available, the free flow of information, interactivity, real-time communication, and opportunities for collaboration [1].

In 2013, Internet use increased to 85% and 72% in the United States and in the European Union, respectively [2]. Besides using personal computers , the rate of mobile phone, tablet, and laptop users also increased underscoring the importance of "continuous online presence" [3,4].

The use of the Internet to search for health-related information has become a common practice worldwide [5]. As such, the online world is a primary source of medical information. Online searches for health-related information doubled between 2002 and 2012 from 40% to 80% [5,6]. Approximately 80% of Internet users search for medical information using search engines, the third most regular online activity after web browsing and checking emails [7].

Besides basic online activities, social media plays an increasingly important role in medical communication [8,9]. For example, 45% of Norwegian and Swedish hospitals are using LinkedIn, 22% of Norwegian hospitals use Facebook for health communication, and statistics reported Facebook as the fourth most popular source of health information [10,11].

The Internet and medicine connect on numerous bases from telemedicine and mobile applications to online practices and community sites affecting the world of research [12,13]. Therefore, digital literacy and the knowledge and use of digital tools in communication, keeping ourselves up-to-date, dealing with information pollution, using mobile health devices and applications, as well as assessing the quality of medical websites has become a crucially important skill in medical practice [14].

The profession of practicing medicine is based on communication, and as social media and other digital technologies play a major role in today’s communication, digital literacy, defined as the ability to effectively and critically navigate, evaluate, and create information using a range of digital technologies, must be included in the medical curriculum [15]. Since the value of social media has been demonstrated several times in medicine and health care, it is time to prepare medical students for the conditions they will have to face when they graduate. In addition to answering traditional medical questions, they will have to be prepared for patients asking questions about the World Wide Web.

Use of social media channels for personal rather than professional purposes, unintentionally revealing details of patients’ private information, and the massive social media use by medical students raise awareness about the importance of acquiring skills related to digital technologies from the first year of medical school [16]. While there have been attempts at using social media platforms in education, examples are sporadic and an extended curriculum is very much needed [17-19].

An online physician study (N=5000) showed that 80% of Hungarian physicians used the Internet on a daily basis, mainly for searching relevant literature or for the drug database. Almost half of them believed that he/she had a worse or same level of knowledge of digital technology than the average population. This suggests that around 50% of the surveyed physicians thought that digital literacy should be introduced to the curriculum of medical education [20].

In order to include the teaching of digital literacy skills in the medical curriculum, an elective course was launched at the University of Debrecen, Medical and Health Sciences Center, in 2008, and later moved to Semmelweis University in 2012. As medical students from around the world demanded access to the materials, an online e-learning platform and a Facebook challenge with gamification were launched in 2012 in order to motivate students to improve their digital skills during the course of the semester.

The aim of this study was to test whether such an interactive, on-and offline-based curriculum with innovative methods could help medical students acquire digital literacy skills, as well as to improve their knowledge about the digital world. Here, we demonstrate the methodology, format, and details of the curriculum. We also aimed at analyzing the descriptive feedback of the student participants. The main educational goals included testing the curriculum, format of the course, and design of the online channels made to teach students about digital literacy through digital channels. Our study, however, did not focus on the long-term consequences of the curriculum; these will be analyzed and discussed in further studies.

## Methods

### Student Demographics

Data of surveys filled in by 932 medical and dentistry students, from over 20 countries, of Semmelweis University in the 2010/2011, 2011/2012, and 2012/2013 semesters were included in this study. Each student who enrolled completed the course modules and filled in the survey.

### Procedure

Students (N=932) attended live lectures for 12 weeks where Prezi-formatted presentations were shown [21]. Students were encouraged to use touch screen and laptop devices during the lectures to improve the digital communication between them and the lecturer. They had the opportunity to ask questions on Twitter as well during the lectures.

A Facebook page was established to launch a competition. The first students to correctly answer the questions covering the topics and learning points of the lectures got bonus points. There were 1-3 questions posted every day during the semester.

The e-learning platform was designed to let the students re-watch the presentations, access all materials and references mentioned in the lectures, and do tests. Completing the online course resulted in an opportunity to skip the written exam. Students had a written test at the end of the semester.

### Online Surveys

Students filled in online surveys before and after the course using the website SurveyMonkey [22] (Multimedia Appendix 1). The survey included questions that allowed students to self-report changes in their digital skills, as well as questions that objectively measured the change in their attitude. The surveys included questions about demographics, digital habits, Internet use, online study solutions, their knowledge of social media, empowered patients, the devices they have, and feedback about the course.

Statistical analysis was descriptive, focusing on percentages and averages. Anonymous data were visualized by Microsoft Excel 2007.

Hungarian regulations such as Decree No 35/2005 (26th of August, 2005) of the Ministry of Health on the clinical trial of investigational medicinal products for human use and on the application of the good clinical practice, and Decree No 23/2002 (9th of May, 2002) of the Ministry of Health on biomedical research on human subjects (applied to conducting a clinical trials) are not applicable in our research, therefore, ethical approval was not required.

### Course Structure

The elective course is an official part of the medical curriculum at Semmelweis University, Budapest, Hungary, both in the Hungarian and English programs. It consists of 10 lectures, each 1.5 hours in length, as well as platforms to openly discussing the content on the course Facebook page, the written exam, and the 2 online surveys.

The lectures were developed based on current global trends of social media use; the first textbook of the area (Social Media in Clinical Practice [23]) and the feedback of global experts such as E-Patient Dave deBronkart, Lucien Engelen, or the Pew Internet Research Project, in order to make sure all the relevant and crucial topics are covered in an extensive but evidence-based manner. The course focuses more on the meaningful use rather than the actual popularity of these channels and methods (Textbox 1).

**Course lecture topics.**

Topics

- Introduction to social media and medicine with practical examples: basics of using the Internet, history of online communication, key social media channels and their evolution, ethical aspects of using the Internet for medical purposes.
- Medical search engines and Google applications in medicine: tips and tricks about doing online searches either on regular search engines or medical/scientific ones. The pros and cons of using Google tools in medicine.
- Being up to date with RSS and online resources in education: methods to deal with information pollution, tracking search results, scientific papers, medical information, and news.
- The mysteries of medical blogging: the advantages and potential dangers of writing a medical blog.
- Using Twitter and other microblogging platforms for medical purposes: description of the quickest online channels and its use in medicine and health care.
- Using medical community sites, tools for online collaboration: rules of using social networks such as Facebook as well as medical ones as medical professionals. Tools to collaborate and write manuscripts online.
- Medical mobile phone (smartphone) applications: how to assess the quality of medical mobile phone (smartphone) applications.
- The world of e-patients and social media in health care: how patients and healthcare institutions can and cannot use social media.
- The medical aspects of Wikipedia and wikis: the power of masses, building communities, and assessing the quality of medical information on Wikipedia.
- Virtual environments in medicine: examples when virtual reality environments are used for collaboration and global communication.
- The future of medicine and social media: future technologies and trends that shape the future of medicine with an emphasis on online communication.

### Design of the E-Learning Platform

**Figure 1:**
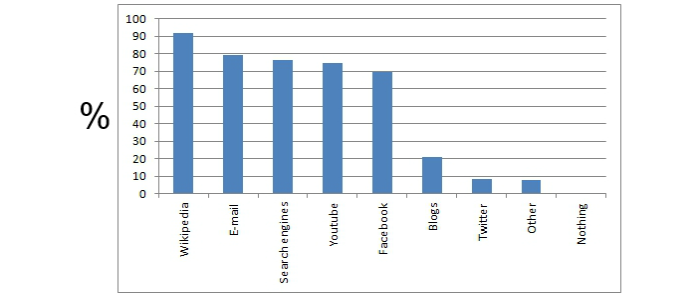
Students enrolled in the course filled in online surveys about the online resources they use in their studies. The y axis represents the percentage of students that chose the particular answer.

The e-learning platform [24] was launched in April, 2012, under the name "The Social MEDia Course" (Figure 1). The course consists of 16 extended presentations in Prezi format with handouts, references, a comment section, and a test for each presentation.

In order to use the concept of gamification in motivating students to continue the study process after the lectures, successfully completing the test as well as completing other tasks resulted in the acquisition of a badge that students could share on their social media channels. Badges were also provided with different levels of activities on the site such as leaving meaningful comments on the presentations, asking questions, or submitting new test questions for future students, thus further motivating them to proceed with the e-learning element of the course. Finishing all the tests earned the "Ultimate Expert" badge and a certification of completing the course.

The platform also includes a community feature which lets students crowdsource challenges related to the digital worlds together [25], and another feature allowing students to submit more challenging questions for the online tests [26].

## Results

### Online Habits

All students had access to the Internet at home or at the university. They used at least one online resource in their studies such as Wikipedia (91.8%, 856/932), email (79.5%, 741/932), search engines (76.3%, 712/932), YouTube (74.4%, 694/932), Facebook (69.5%, 648/932), blogs (21.0%, 196/932), Twitter (8.1%, 76/932), and other (7.7%, 72/932) (Figure 1).

**Figure 2:**
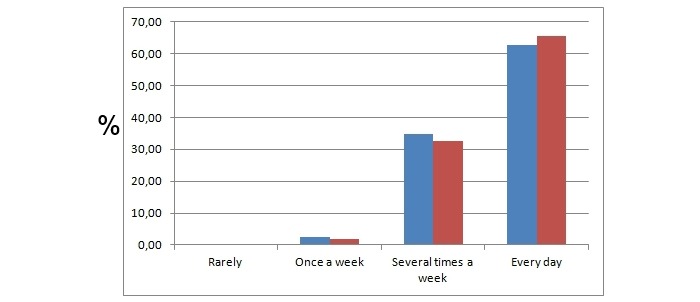
Internet usage of the students enrolled in the course. The y axis represents the percentage of students that chose the particular answer. Blue and red bars represent responses acquired before and after the course, respectively.

The vast majority of the students used the Internet on a daily basis before (62.6%, 583/932) and after (65.4%, 609/932) the course, and this did not increase due to the course (Figure 2).

### Acquired Skills

**Figure 3:**
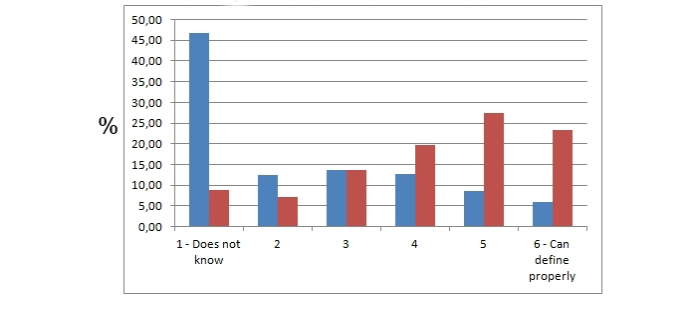
The number of students that could properly define social media. The y axis represents the percentage of students that chose the particular answer. Blue and red bars represent responses acquired before and after the course, respectively.

**Figure 4:**
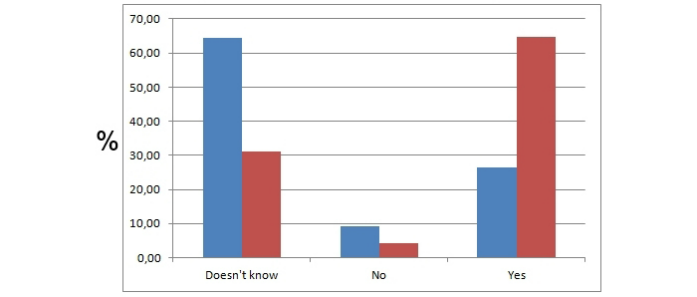
Whether the students believe they will meet the special needs of e-patients. The y axis represents the percentage of students that chose the particular answer. Blue and red bars represent responses acquired before and after the course, respectively.

Based on the survey results, students learned how to define social media, medicine 2.0, health 2.0, e-patients, blogs, RSS, and Twitter (Figure 3). The percentage of those responders who did not know how to define social media dropped from 46.7% (435/932) to 8.8% (82/932), and those who could define it perfectly increased from 5.8% (54/932) to 23.2% (216/932) during the course. They learned how to assess the quality of medical websites, how to transform frustrated patients to e-patients who could be equal partners, and what websites they could share with their future patients. Students thought they could meet the needs of e-patients in their professional lives and would meet their expectations (Figure 4).

### Feedback

**Figure 5:**
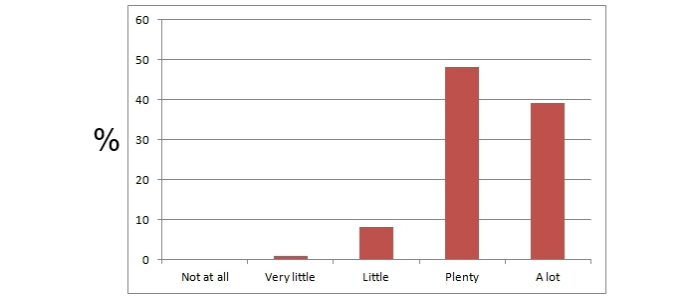
Students enrolled in the course filled in online surveys about how many useful suggestions the course provided them with. The y axis represents the percentage of students that chose the particular answer.

**Figure 6:**
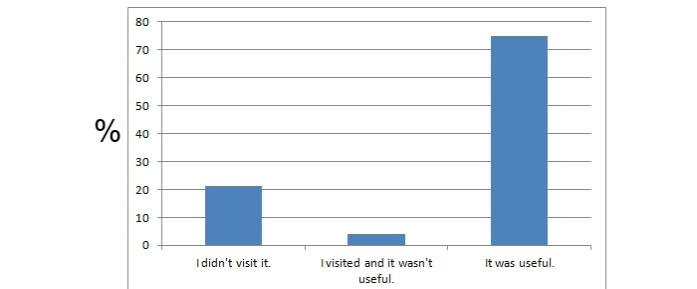
The number of students that visited the course website. The y axis represents the percentage of students that chose the particular answer.

The suggestions provided by the course were valued as highly valuable and quite valuable, and overall, 99.7% (929/932) of students liked the course (Figure 5). Most of the students (74.7%, 696/932) also visited the website and found it useful (Figure 6).

## Discussion

### Principal Findings

This is the first example demonstrating that digital literacy, with a special emphasis on social media describing how major platforms such as Facebook, blogs, Youtube, Wikipedia, Twitter, and Google, can and should be used in the medical profession, and was successfully added to an official medical curriculum (offline and online).

Several methods including gamification were designed to reach and teach students where they spend their online time. As all the students had Facebook accounts, challenges, tasks about online activities, and questions about the topics covered during the lectures were posted every day during the semester on the course Facebook page [27] and the student with the most bonus points did not have to take the written exam.

Students learned new skills during the lectures which focused on practical examples and real-life situations, and they accessed the content in an e-learning platform where they were motivated with features implementing gamification.

### Limitations

A limitation of this methodology might be the lack of access to the Internet in other areas, and the small numbers of cases of e-patients or medical professionals describing the use of social media on a national level as mostly global examples are available. A possible reason behind the observation that 21.3% (199/932) of survey responders did not visit the course website and 3.8% (36/932) of them visited but did not find it helpful might be that students prefer watching videos on e-learning platforms rather than watching presentations, reading studies, and doing tests. Another reason might be that no other course at the medical school uses such innovative methods including the interactive e-learning platform or Facebook challenges in teaching, therefore, time might be required for students to get accustomed to these techniques.

As this is the first study focusing on whether digital literacy skills can be added to a course at a medical school and whether these skills can be improved upon during the course, not being able to compare these results to other studies was also a limitation.

### Conclusions

We believe that a well-designed course improved with constant evaluation-based feedback is suitable for preparing students for the massive use of the Internet, including social media platforms and digital technologies, and new approaches must be applied in modern medical education in order to teach students skills required for a world rich in digital technologies and e-patients.

Courses worldwide provide curriculums about medical informatics and information retrieval which have become an important part of training medical students [28-31]. To the best of our knowledge, this is the only course in the official medical curriculum that not only implements social media channels in teaching certain skills but also focuses on the use of the digital world, especially social media platforms, for medical and other professional purposes. This study was meant to raise awareness to the importance of introducing medical students to the use of digital solutions in the settings of medical education and generate a discussion about what other methods could also be used to reach this long-term goal.

The structure of this course appears to be an efficient way of transforming medical students’ views about the Internet, show them professional behavior, teach them how to create online profiles for themselves, and how to get to a meaningful use of a huge range of online tools and platforms. Students require specialized training in order to fill health care with technology-savvy professionals.

## Appendix

Multimedia Appendix 1 Survey questions.
