# Supplementary material for: Digital Literacy in the Medical Curriculum: A Course With Social Media Tools and Gamification
Source: JMIR Med Educ. 2015 Oct 1;1(2):e6. doi: 10.2196/mededu.4411 (PMC5041363; doi:10.2196/mededu.4411)
Supplement: Multimedia Appendix 1 [file mededu_v1i2e6_app1.pdf]

Social Media in Medicine survey 2014-15

It will only take a few minutes to complete the survey. I greatly appreciate your time!

Please answer the questions as if we were before the course!

*** 1. What is your e-mail address?**

*** 2. What is your FULL NAME as registered in Neptun? (First name, then Last name)**

*** 3. What is your age?**

*** 4. What is your gender?**

- Male
- Female

*** 5. What is your first or best spoken language?**

- English
- Spanish
- Chinese
- French
- German
- Korean
- Russian
- Italian
- Arabic
- Other
- Other (please specify)

*** 6. In which year are you currently in regarding your studies?**

- First
- Second
- Third
- Fourth
- Fifth
- Sixth

***7. Where do you study?**

- Faculty of Medicine
- Faculty of Dentistry
- Faculty of Pharmacy
- Other (please specify)

***8. How often do you use the Internet?**

	Never	Rarely (once a month)	Once a week	Several hours a week	Once a day	Several hours a day
Please choose	○	○	○	○	○	○

***9. Do you use online tools in your studies?**

○ Yes

○ No

***10. If yes, what kind of websites do you use? (You can select more answers)**

☐ Blogs

☐ Wikipedia

☐ Second Life

☐ Search engines

☐ E-mail

☐ Facebook

☐ Twitter

☐ Youtube

☐ I don't know these.

☐ Other (please specify)

***11. How important role do you think the internet can play in the future of medicine? (1 means it has no role and 6 means it will play a very important role)**

	1	2	3	4	5	6
Please choose a number	○	○	○	○	○	○

***12. Have you ever heard about web 2.0 or social media?**

- Yes
- No

***13. If yes, what kind of role web 2.0 can play in your studies or in your practice?**

***14. Could you define web 2.0 or social media? (1 means I don't know what web 2.0 is and 6 means I could define it and tell examples as well)**

	1	2	3	4	5	6
Please choose a number						

***15. Have you ever heard about medicine 2.0 or health 2.0?**

- Yes
- No

***16. Does medical education meet the challenges of the 21st century? If it doesn't, what are the main problems with it?**

***17. Have you ever heard about e-patients?**

- Yes
- No
- I don't know.

***18. Do you think you will ever meet an e-patient and you will be able to meet their expectations?**

- Yes
- No
- I don't know.

***19. What kind of websites would you show to your patients?**

- Sites accredited by HONcode.
- Sites accredited by HBCE.
- Sites using HIPAA.
- I don't know these

*20. Would you communicate with patients through these tools?

- [ ] E-mail
- [ ] Facebook
- [ ] Twitter
- [ ] Blog
- [ ] I wouldn't

*21. What do you think about the growing popularity of internet?

	I don't know what web 2.0 is	It scares me.	It worries me.	I'm neutral	I find it interesting	I find it exciting
Please choose a number	◯	◯	◯	◯	◯	◯

***22. Which of the following social network sites do you use?**

- [ ] Facebook
- [ ] MySpace
- [ ] LinkedIn
- [ ] Patientslikeme
- [ ] Other (please specify)

***23. Do you read medical blogs?**

	I don't know	Never	Rarely (once a month)	Once a week	Once a day	Several times a day
Please choose a number	○	○	○	○	○	○

***24. Do you use RSS readers/aggregators? (e.g., Feedly)**

	I don't know	Never	Rarely (once a month)	Once a week	Once a day	Several times a day
Please choose a number	○	○	○	○	○	○

***25. Do you use microblogging applications? (e.g., Twitter, Friendfeed)**

	I don't know	Never	Rarely (once a month)	Once a week	Once a day	Several times a day
Please choose a number	○	○	○	○	○	○

***26. Where did you hear about this course?**

- ○ E-mail
- ○ Bulletin board
- ○ Friends
- ○ Professors
- ○ Blogs
- ○ Other (please specify)

***27. What do you have? (You can select more)**

- [ ] Laptop
- [ ] Personal computer
- [ ] Mobile phone
- [ ] Smart phone
- [ ] Digital camera
